# Traditional Mongolian, Traditional Chinese, and Western Medicine Hospitals: System Review and Patient Survey on Expectations and Perceptions of Quality of Healthcare in Inner Mongolia, China

**DOI:** 10.1155/2018/2698461

**Published:** 2018-07-19

**Authors:** Min Li, Yancun Fan, Edward B. McNeil, Virasakdi Chongsuvivatwong

**Affiliations:** ^1^Epidemiology Unit, Faculty of Medicine, Prince of Songkla University, Songkhla 90110, Thailand; ^2^Faculty of Health Management, Inner Mongolia Medical University, Hohhot 010020, China

## Abstract

**Background:**

In Inner Mongolia of China, traditional Mongolian medicine (TMM), traditional Chinese medicine (TCM), and western medicine (WM) are all supported by the government. This study compares the background and performance of these three types of medicines.

**Methods:**

The World Health Organization's Six Building Blocks framework was used for the system review. Data were collected from literature review and key informant interviews. A cross-sectional survey was conducted in three types of hospitals at the provincial, municipal (city), and prefectural (county) levels from April to August, 2016. Eight hospitals were included and, within each one, patients from four outpatient departments were selected. A total of 1,322 patients were interviewed about their expectations and perceptions of the health service.

**Results:**

Government support for TMM includes higher budget allocation and a higher reimbursement rate. TMM is preferred by Mongolian people, those living in pasturing areas, and those seeking treatment for musculoskeletal problems/injuries. Patients attending TMM hospitals had the highest expectations and perceptions of the health service in general. However, human resources and research capacity of TMM are relatively limited.

**Conclusion:**

To further enhance the role of the popular TMM for local minority's health, human resources and research capacity strengthening are essential.

## 1. Introduction

China is a multiethnic country, containing no fewer than 56 ethnic minority groups. Inner Mongolia Autonomous Region, which is the most important settlement of Mongolian people in China, was the first established minority autonomous region of China. There are approximately 24 million people living in Inner Mongolia hosting 49 minority groups, the majority of which is the Mongol ethnicity.

Traditional medicine is an indigenous medicine that is used to maintain health and to prevent, diagnose, and treat physical and mental illnesses [1]. Traditional Chinese medicine (TCM) has been widely used in China for the past 3,000 years [2]. In Inner Mongolia, traditional Mongolian medicine (TMM) has been recognized as an important part of the healthcare system. It is a systematic theoretical system and has abundant resources of medicaments and special therapies [3]. It is an important component of the Chinese national medicine system and contributes a significant part to the wealth of world traditional medicine [4]. The three types of medicine are distributed uniformly across the province.

Research on TMM has mostly been confined to basic science and development of TMM products. Seldom research considered to review and describe the TMM health system, as well as evaluate the performance of health systems from the patient's perspective. In order to gain a better understanding of TMM, this study examined the national and Inner Mongolia health system in relation to TMM and evaluated the health system performance by patient survey. This will broaden consideration of TMM stakeholders to health system aspects. The objectives of this study are to review the health system related to TMM and to evaluate the health system's performance on the basis of patients' expectations and perceptions on the service they received from TMM hospitals compared with TCM and WM hospitals. The study was divided into two parts: a system review and a hospital-based patient survey.

## 2. Methodology

### 2.1. System Review

We based our review on the World Health Organization (WHO) six building blocks framework [5] which consists of (i) service delivery; (ii) health workforce; (iii) information; (iv) medical products and technologies; (v) financing and sustainability; and (vi) leadership and governance. These blocks of TMM were compared with those of TCM and WM. We also considered process elements (access, coverage, quality, and safety) and outcomes (improved health and health equity, responsiveness, social and financial risk protection, and improved efficiency).

In the literature review keyword search terms included “traditional Mongolian medicine”, “traditional Mongolian medicine health system”, or “traditional Mongolian medicine and traditional Chinese medicine”. Source of information included domestic and foreign papers, books, meeting reports, dissertations, publicly accessible government data, and national statistics. We also made use of data based on the health workforce and financial investments [6].

With a well-prepared guideline in Supplementary Materials (available here), we conducted in-depth interviews with key informants, including policy makers, presidents of the schools, and doctors and managers of TMM hospitals, to collect their perception of the TMM health system. Interviews were conducted by the principal investigator and two well-trained research assistants.

The decentralized information obtained by literature review and key informants interview was classified by using MAXqda.11 software and summarized by using the six building blocks framework. The comparison of six aspects across different types of medicine was shown in a concentrated table and description. The results were discussed under the evidence provided by patient survey.

### 2.2. Patient Survey

A cross-sectional survey was conducted in two cities: (1) Hohhot: the capital of Inner Mongolia, and (2) Baotou: a major industrial city. These two cities were chosen because they got enough number of hospitals and patients to allow our detailed comparison. Eight hospitals, four from Hohhot and four from Baotou, were chosen for the survey, including four TMM hospitals, two TCM hospitals, and two WM hospitals. We overselected TMM hospitals as TMM is the focus of this study. We selected hospitals from high (provincial) level to low (county) level to improve the generalizability of our results, to facilitate comparison between hospitals of different levels, and to achieve the required sample size in a timely fashion. Therefore among the TMM hospitals, one provincial level, one city-level, and two county-level hospitals were selected. The WM hospitals were all at the provincial level while, among the two selected TCM hospitals, one was a provincial level and one a city-level hospital.

For comparability across hospitals and to obtain a broad group of clients, in each selected hospital we sampled patients from four major departments, namely, the departments of gynecology, pediatrics, and orthopedics and cardiovascular department. All outpatients who were able to communicate, read, and write in either Chinese or Mongolian were eligible for the study. Exclusion criteria were cognitive or memory dysfunction, or severe mental illness, such as schizophrenia and serious depressive disorders.

We devised the questionnaire collecting data on socioeconomic characteristics and disease-related information. The part measuring patient's expectations and perceptions was adopted from a revised Chinese version of the SERVQUAL model [7] shown in Supplementary Materials, which has been well validated and widely used in China. In brief, this model assesses clients' evaluation based on the difference between their expectation and perception on health service. It consists of two parts, the patients were first asked about their expectations and then asked to state their perceptions. Both parts share the same set of 28 statements covering seven domains: tangibles, reliability, trust, responsiveness, humanized service, effectiveness, and economy. The level of patients' agreement with each statement is rated on a scale ranging from 1 (least agree) to 9 (most agree).

Patient interviews, using the above questionnaire, took place between April and August 2016 by 12 third-year medical students who were specially trained and employed as research assistants. The venue was the hall where the patients were waiting to pay or to have further tests. Eligible subjects were identified and approached by the nurses who explained the aims of the study and consent was requested from the patients if they were older than 18 years or from their parents/guardians otherwise. Interviews, each lasting approximately 30 minutes, took place in a quiet and private location near the waiting area.

The sample size was calculated using the comparison of two means formula as follows [8, 9]:

(1) n1=z1−α/2+z1−β2σ12+σ22/rΔ2r=n2n1,Δ=μ1−μ2

Using a type I error of 5% and a power of 80% to detect a difference in mean gap scores of 0.06 between two groups of patients and a standard deviation of 0.06 from a previous study [10], 35 patients were required in each hospital department. A design effect of 1.2 was used in order to compensate for similarity of the outcome variable within patients of the same hospital. Finally, the study included 1,344 patients as research. Finally, 1322 questionnaires are valid.

Data were double-entered in Epidata version 3.1 and analysed using R version 3.1.2 [11]. Patients' background information was summarized using descriptive statistics. The scores were analysed in two aspects: (1) expectation scores were subtracted from perception scores of the same individual yielding a gap score, (2) comparisons of expectation, perception, and gap scores across different types of medicine and different levels of hospitals were conducted. Boxplots were used to display the results. Analysis of variance was used to test differences in service quality across groups. The relationships between the patient's expectation, perception, and gap scores with the three hospital types and levels were determined using multivariate linear regression models after adjusting for patients demographic, socioeconomic, and disease-related factors.

## 3. Results

### 3.1. Results of System Review

Table 1 compares major aspects (modified from WHO's six building blocks) of TMM, TCM, and WM. The entries in the first column of the table correspond to the following headings.

*History*. The inheritance of TMM in Inner Mongolia was much later than the origination of TCM in China whereas WM was introduced many centuries later. Formal training of the medical profession in all three types started after the 1949 revolution [12]. Rapid progress in regulation was made after the turn of the millennium when China's economy strengthened.

*Service Delivery*. TMM and TCM have a similar small share in service delivery. The health service provided by WM hospitals is around three times that of TMM and TCM hospitals combined. WM is the backbone of health services since it covers all levels of health care.

*Health Workforce*. Despite policy to accelerate training of TMM and TCM health personnel during the last 10 years, currently, the combined health workforce is still only one-third that of WM. The two types of traditional medicines combined also have a smaller workforce training capacity and productivity than those of WM. To compensate for the shortage, apprenticeship training for TMM is still formally accepted [24]. However, trainees have to pass the license examination before being allowed to provide medical services and the pass rate for TMM candidates is lower [35].

*Health Information System*. All types of hospitals have to submit their basic information, including institutions, facilities, health workforce, and health service, to the unitized medicine information system [37]. The TCM and TMM administrations in Inner Mongolia have the responsibility of producing, analysing, disseminating, reporting, and using this information related to TMM. A TMM literature information center is located in Inner Mongolia Medical University and is responsible for the collection, collation, and publication of TMM literature. The government is currently building a TMM information system with unified translation standards of minority languages [37]. This may or may not be similar to TMM in Mongolia and other countries where people of Mongol ethnicity reside.

*Medical Products and Technologies.* The numbers of medicaments within each of the three types of medicines are comparable. TMM has its preeminent performance in the field of orthopedics, which was largely necessitated by horse riding and nomadic lifestyles of the people [13]. Production of the TMM industry was limited by substandard research, which was much slower than for TCM. Its market share was only 0.75 percent of TCM and 0.15 percent of WM [43]. Most of the effective TMM medicaments and formulations were abandoned in the standardization [47]. Medicaments and formulations with standard licenses produced by companies were refused by TMM hospitals. On the other hand, formulations produced in TMM hospital laboratories are not allowed by the government to be sold to the public.

*Financing and Sustainability.* TMM hospitals have one-third higher revenue than their TCM counterpart. WM hospitals, however, have revenue nearly four times higher than the other two combined. TMM hospitals are heavily subsidized in terms of budget allocation, staff salaries, and reimbursement from the health insurance system. However, the number of items reimbursable is less than 10% of those in TCM and WM hospitals.

*Leadership/Governance.* Responsibilities of TMM and TCM administrators include provision of special policies and development planning on the basis of the principles of national policies and the health situation of people in Inner Mongolia [22]. Management functions include designing the system framework and designing the standards and drugs catalog, guidance, and oversight, which are all unified to facilitate better coordination [22]. On the basis of these responsibilities and functions, the government can better promote the development of TMM, protect the Mongolian culture, and provide equal rights for all ethnic minorities to develop. However research projects supporting TMM and TCM have mainly been biomedical (basic science) in nature whereas clinical trials and public health are mostly confined to WM.

### 3.2. Results of Patient Survey

The basic characteristics of the study hospitals are shown in Table 2. Four provincial hospitals were selected from Hohhot city, two offering western medicine and one each offering traditional Chinese medicine and traditional Mongolian medicine. The four hospitals from Baotou city, two city-level and two county-level, all offered traditional medicine.

A comparison of characteristics of respondents attending Western medicine, traditional Chinese medicine, and traditional Mongolia medicine hospitals is shown in Table 3. The study sample had mean age of around early thirties and was predominated by females (63%). Higher ratio for female was due to the fact that one of the sample departments was gynecology. The TMM hospitals had higher proportions of older patients, Mongolian people, patients with lower education, and residents living in pasturelands. TCM hospitals had a higher proportion of poorer patients and those not currently employed. Patients attending WM hospitals were more educated and tended to reside in a different locality from the hospital where they sought treatment. The distributions of marital, occupational, and insurance status were more or less balanced.

On clinical aspects, four groups of diseases (cardiovascular diseases and diseases of the blood, musculoskeletal problems and injuries, diseases of the genitourinary system and gestation, and diseases of the respiratory system) accounted for 83% of all study patients. The distribution of diseases by medicine type was generally balanced except that TMM hospitals had a relatively higher proportion of patients with musculoskeletal problems and injuries, TCM hospitals had a higher proportion of patients with diseases of the genitourinary system, and WM hospitals had a higher proportion of patients with cardiovascular, blood, and respiratory system diseases. TCM hospitals tended to have a higher proportion of established patients. In general, the majority of the patients visited the hospitals for the first time and with symptoms lasting for 4-30 days. The quality of health service was the main reason for choosing the particular hospital among all patients.

**Figure 1:**
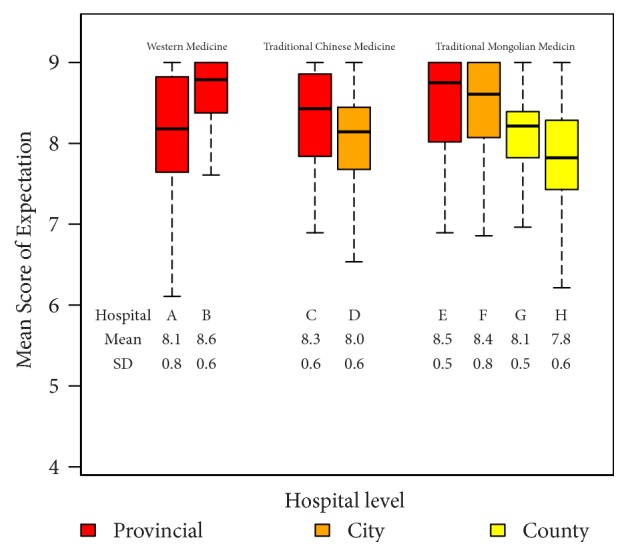
Comparison of patient's expectations of health services by type and level of hospital (N=1,322).

Figure 1 shows a comparison of patient's expectations of the health services by type and level of hospital.

The median expectation scores for all hospitals ranged from 7.8 to 8.8 and the overall mean expectation score was 8.2. Patients attending WM hospitals had a higher expectation than those attending TCM and TMM hospitals (*p* = 0.001). Patients who attended provincial level hospitals had the highest expectation, followed by city-level and county-level hospitals. There were also variations in expectation within hospitals offering the same type of medicine, particularly TMM hospitals.

**Figure 2:**
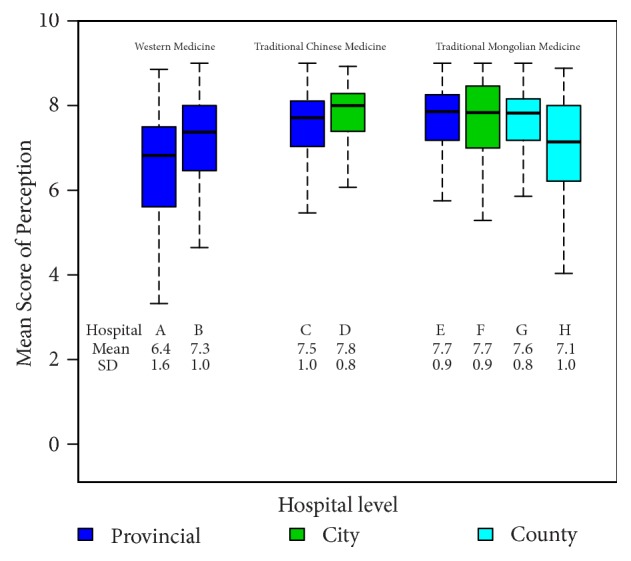
Comparison of patient's perceptions of health services by type and level of hospital (N=1,322).

Figure 2 shows a comparison of patient's perceptions of the health services by type and level of hospital.

Patient's perceptions of the quality of healthcare services differed from that of their expectations. Patients attending WM hospitals had a lower perception compared to patients attending TCM and TMM hospitals (*p* <0.001). Among the TCM and TMM hospitals, patients attending city-level hospitals had a higher perception of health services than the others.

**Figure 3:**
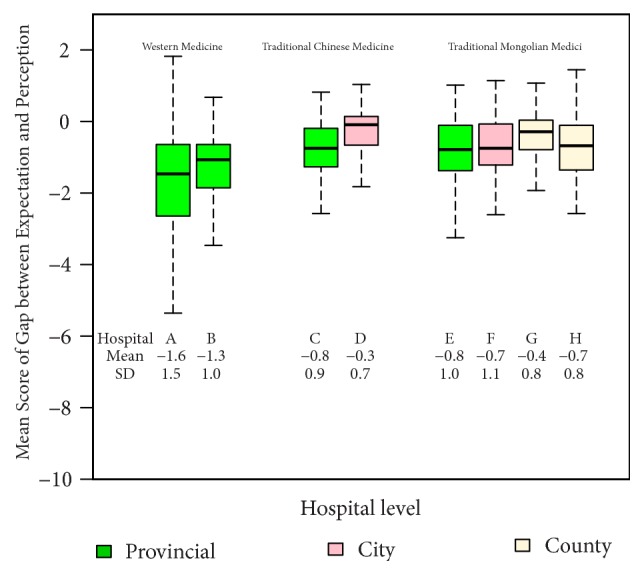
Comparison of patient's gap scores by type and level of hospital (N=1,322).

Figure 3 shows a comparison of the difference between patient's expectations and their perceptions of the health services (the gap score) by type and level of hospital. A negative value indicates that patient's perceptions were below their expectations. Due to relatively high expectation scores, most hospitals had negative gap scores. The largest negative gap score was seen in WM hospitals (mean difference: -1.6). TCM and TMM hospitals had a median difference of zero indicating that patient's perceptions tended to match their expectations.

**Figure 4:**
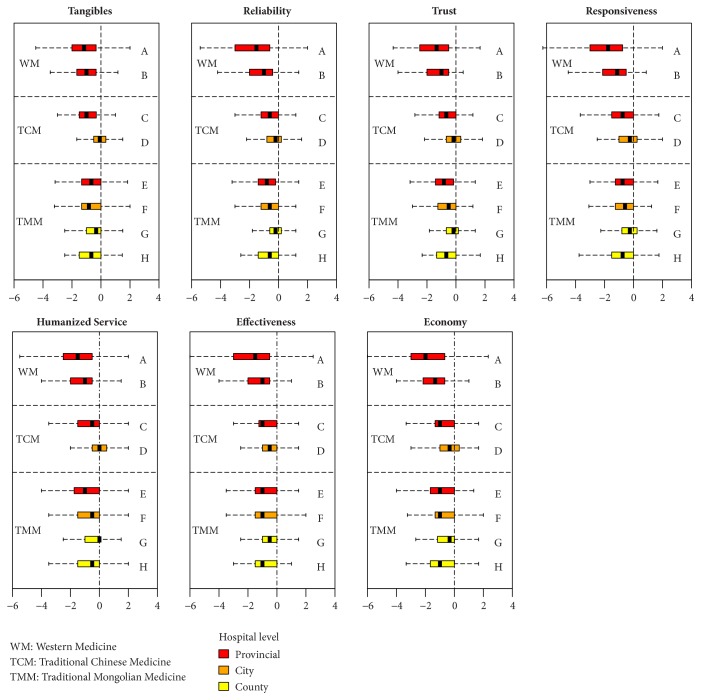
Comparison of patient's gap scores for quality of service domains by type and level of hospital (N=1,322).

Figure 4 compares the gap scores for the seven quality of service domains stratified by hospital type. Median gap scores for all domains were less than zero, with WM hospitals having noticeably lower scores compared to the other hospitals. Health service perceptions of patients attending city-level TCM hospitals were higher than zero for most domains.

Table 4 shows adjusted coefficients and their 95% confidence intervals of factors from the final linear regression models predicting patients' expectations, perceptions, and gap. The models included 12 variables, but only results for medicine type and hospital level, the main focus of this analysis, are presented.

Patients attending TMM hospitals had the highest level of expectation and perception; however, their overall evaluation of the health service quality (the gap scores) was lower than patients attending TCM hospitals. Patients attending WM hospitals were the most dissatisfied with the quality of the health service.

## 4. Discussion

Data from health information systems indicated that TMM plays important roles in health services. It is vibrant and accepted by the minority groups with preeminent performance on orthopedics. Compared with TCM and WM, health service capacity of TMM was limited by a less number of hospitals, less health workforce, weaker educational capacity, and behindhand research. However, it receives a higher subsidy from regular government investment and reimbursement schemes.

Our survey significantly assists our understanding of how local people perceive the three health systems. It revealed the popularity of TMM among respondents who were of Mongol ethnicity and living in pasturelands. The survey also highlights more important roles of TMM in serving musculoskeletal problems of outpatients. Our study revealed that the clients of TMM hospitals had the highest level of expectation and were also more likely to have higher perceptions than clients of the other two hospital types.

The regular utilization of traditional medicine as a component of a health system is accepted by half the population of many developed and developing countries [26, 48–53]. In our study setting, traditional medicine is not only popular but also strongly supported by the government. The proportion of services provided by TMM in Inner Mongolia is more substantive than that of Thai Traditional medicine in Thailand but smaller than Ayurveda in India [54, 55].

Coexistence of the three medicine subsystems in the same community may initiate competition for patients between the hospitals, especially in a small service market [22]. It also forms a competitive relationship in resources, especially human resources. The large hospitals will capture health personnel working in TMM or TCM hospitals due to their financial strength. This mobility of health workforce usually happens from lower level to higher level hospitals. As a result, inadequate health services in rural China [56] are therefore not confined to WM but also traditional medicine.

The prevailing dominance of WM over traditional medicines in China, including TCM, is seen in most of its provinces as well as in other countries [27, 57, 58]. With advanced technology, the expectations of the majority of patients attending WM hospitals are relatively high. Their overwhelming numbers of patients and inadequate subsidy from the government also fuel the deteriorating doctor-patient relationship [59] which is evidenced by their low perception scores in our study. This weakness in the mainstream health system needs to be corrected.

Not all people who are ill need to see a doctor. Self-treatment for minor ailments with safe and effective over-the-counter products can be useful in many cases and it is a common practice worldwide [60]. On one hand, TCM products, which often do not have proof of safety or efficacy, are heavily advertised on public media. On the other hand, TMM products have a very narrow clientele due to poor scientific research and poor market mechanisms. These deficiencies need to be addressed by policy-makers. To promote TMM, it is therefore necessary to incorporate scientific research into TMM to improve its efficacy and safety. This would further strengthen pride and acceptance of local people.

This study showed that patients' expectations of health service quality are higher than their perceptions, a result seen in other studies from Iran [61], Turkey [62], and Taiwan [63]. However, on multivariate analysis, patients visiting traditional medicine hospitals had positive gap scores, indicating that the health service quality of these hospitals was higher than the western medicine hospitals. Traditional medicine has the potential to improve the overall quality and become an important supplement to Western medicine, especially on humanized service aspects.

Finally, traditional medicines, which are rooted in indigenous medical practice, have accumulated a large number of drugs and contain valuable experience. There have been many successful cases for western medicine to find new ideas, treatments, and drugs from the rich resources of traditional medicine [47, 64]. If traditional medicine systems are lost, countries, especially developing ones, will lose their self-reliability, their culture, and their identity [65].

## 5. Conclusion

In Hohhot and Baotou cities of Inner Mongolia, Traditional Mongolian medicine is well accepted by the government and citizens. It has the potential to improve its overall quality and become an indispensable supplement in the whole health service system. Continued protection and promotion of its development by investment, government policies, education, and biomedical and health systems research will be needed.

## Figures and Tables

**Table 1:** Comparison of the TMM health system with TCM and WM health systems in Inner Mongolia based on the six building blocks framework.

**Building block**	**Details**	**Traditional Mongolian medicine **	**Traditional Chinese medicine**	**Western medicine**	**References**
History	Inherited by or introduced in Inner Mongolia	Inherited by Mongolia between the 13th and 16th centuries.	Originated in China about 3,000 years ago.	Introduced into China and Inner Mongolia in the early 19th century.	[12–16]
	Establishment of the first hospital	1940s	1900s	1830s	[12, 14, 15]
	Year of establishment of the first faculty (start of undergraduate program)	1958	1958	1956	[12, 17–19]
	Regulations	“Regulation of TMM and TCM in Inner Mongolia”, the first local laws and regulations about traditional medicine came in 2001. The new version came in 2010.	“Regulation of TCM of People's Republic of China” came in 2003.	Drug Administration Law of China in 2008. Regulations of Medical Institutions in 2005. Regulations of Medical Accidents in 2008.	[14, 20, 21]

Service delivery	Percentage of hospitals providing this kind of medical service in 2015	16%	19%	74%	*∗*
	Number of beds in 2015	15,649	14,146	84,280	*∗*
	Level of coverage	Partial. In some areas, township and village levels are covered by neither TMM nor TCM		Complete (all levels)	*∗*
	Number of outpatients in 2015 (million)	7.6	6.3	34	*∗*
	Number of inpatients in 2015 (million)	0.3	0.3	2.1	*∗*
	Clinical department	Modern medical science department classification; some TMM hospitals have a special clinical section, such as TMM therapy section.			[22]
	Language in service	Chinese or Mongolian	Chinese	Chinese	[23]
	Diagnostic and treatment process	Requires a deep communication with patients.		Accurate diagnosis by using advanced medical equipment and laboratory tests.	[24, 25]
	Acceptance by people ^*∗∗*^	Generally accepted by most minority groups, 25% of Han people never used or never heard of TMM.	Accepted by most people but seen as a supplement of WM.	Accepted by most people as the main health service provider.	[26–28]

Health workforce	Health workforce in 2015 (thousand)	17	15	101	*∗*
	Workforce per thousand population in 2015	0.7	0.6	4.05	*∗*
	Educational model	(i) University only. (a) 5-year Bachelor. (b) 3-year Master or standardized training (ii) Alternative apprentice training		(i) University only. (a) 5-year Bachelor (b) 3-year Master or standardized training.	[24, 29, 30]
	Number of schools	2	2	4	[31–34]
	Number of undergraduate students per year	500	500	1200	[17–19, 31–34]
	Pass rate of National Practicing Physician Qualifications Test	60%	60%	62%	[35]

Health information system	Information for planning, monitoring, and evaluation	By Health and Family Planning Commission and TCM and TMM Administration.		By Health and Family Planning Commission and Public Hospital Reform Office.	[36]
	Health information system outside the National Health Information System	Under the support and guidance of Health and Family Planning Commission; TMM and TCM information systems, including a unified translation standards of minority languages.		Under the support and guidance of Health and Family Planning Commission and Public Hospital Reform Office.	[36, 37]
	Library system	Library of Inner Mongolia University National network.		National and international medical network of electronic link.	[36, 37]

Medical products and technologies	Number of medicaments used in China	More than 2,200 kinds, in which 1,342 are regularly used.	More than 2,500 kinds, including twelve large varieties of prescriptions and preparations.	More than 2,740 medical products normally used.	[22, 38]
	Evaluation of medicine	Good effect on many kinds of diseases, preeminent performance on orthopedics.	Becoming more prominent in the growing challenge of noncommunicable diseases. Slow in action but more thorough in “curing the root of the problem” with few side effects.	Good at diagnosis and treatment, especially for serious or urgent diseases, considered to be “more powerful and quick” but may cause significant side effects.	[22, 26, 39]
	Standardization	In 1986, 322 medicaments were put in the Medicine Standard of Inner Mongolia but only 57 in 1998.	Already had medicaments standard in China. Three medicaments were approved by the FDA to undergo further clinical trials before 2016.	FDA approved more than 10,000 drugs.	[12, 40–42]
	Market value (RMB)	Small market share with 3 billion yuan in 2010.	400 billion yuan in 2005 across the whole country.	More than 2,000 billion yuan in 2015 across the whole country.	[22, 43, 44]

Financing and sustainability	Total revenue in 2015 (million RMB)	4.8	3.6	30.2	*∗*
	Government subsidy (%)	27.5	24.7	13.7	*∗*
	Staff cost paid by the government (%)	100	80	0	[22]
	Proportion of reimbursement from health insurance from basic level to high level	Varies from 80%-30%		Varies from 60%-20%	[22, 45]
	Number of items listed in the Medicare Insurance Reimbursement Catalog 2017	88	1,238	1,297	[46]

Leadership/ governance	Administrative department of Health and Family Planning of Inner Mongolia	TMM and TCM Administrations were set up in the provincial and municipal levels from 2007.		Medical Reform Office Public Hospital Reform Office.	[22]
	Items of autonomy of the Inner Mongolia Autonomous Region Government	Establish the standard for TMM drugs, technology, and hospitals. Establish and negotiate health insurance reimbursement catalog.	Province-specific health insurance reimbursement catalog.		[21]
	Research promotion priority	Biomedical	Biomedical	Clinical and Public Health, Public Hospital Reform	[22]

^a*∗*^Calculated from Health Information Reporting System of Inner Mongolia in 2015.

^b*∗∗*^See Table 3.

^c^WM: Western medicine; TCM: Traditional Chinese medicine; TMM: Traditional Mongolian medicine; TM: Traditional medicine;

IMMU: Inner Mongolia Medical University; FDA: Food and Drug Administration

**Table 2:** Summary characteristics of the study hospitals.

**City**	**Level**	**Sample hospital**	**Type of medicine offered**	**Number of outpatients per year**	**Number of study subjects**
Hohhot	Provincial	A	WM	1,431,420	165
		B	WM	1,369,180	168
		C	TCM	279,910	163
		D	TMM	534,460	163
Baotou	City	E	TCM	48,940	168
		F	TMM	147,870	167
	County	G	TMM	99,340	163
		H	TMM	35,600	165

^a^WM: Western medicine; TCM: Traditional Chinese medicine; TMM: Traditional Mongolian medicine.

**Table 3:** Demographic characteristics of study sample (N=1,322).

	**WM (N=333)**	**TCM (N=331)**	**TMM (N=658)**
Gentle			
Male	119 (35.7)	116 (35.1)	255 (38.8)
Female	214 (64.3)	215 (64.9)	403 (61.2)

Age (mean, SD)	32.2 (21.2)	31.9 (21.0)	34.3 (22.5)

Nationality			
Han	282 (84.7)	297 (89.7)	518 (78.7)
Mongolian	44 (13.2)	23 (6.9)	131 (19.9)
Others	7 (2.1)	11 (3.3)	9 (1.4)

Completed education level			
Primary school or less	111 (33.4)	122 (36.9)	280 (42.6)
Secondary school	110 (33.0)	119 (36.0)	236 (35.9)
Diploma or higher	112 (33.6)	90 (27.1)	142 (21.5)

Marital status			
Single	137 (41.1)	123 (37.2)	237 (36.0)
Married	180 (54.1)	194 (58.6)	401 (60.9)
Others	16 (4.8)	14 (4.2)	20 (3.1)

Occupational group			
None	161 (48.3)	183 (55.3)	332 (50.5)
Private	88 (26.5)	91 (27.5)	208 (31.6)
Public	84 (25.2)	57 (17.2)	118 (17.9)

Insurance status			
None	17 (5.1)	33 (10.0)	53 (8.1)
UEBMIS	112 (33.6)	70 (21.1)	156 (23.7)
URBMIS	73 (21.9)	99 (29.9)	142 (21.6)
NRCMIS	96 (28.9)	105 (31.7)	275 (41.8)
Others	35 (10.5)	24 (7.3)	32 (4.8)

Monthly family income			
None	66 (19.8)	126 (38.1)	150 (22.8)
≤ 4000	149 (44.8)	143 (43.2)	377 (57.3)
> 4000	118 (35.4)	62 (18.7)	131 (19.9)

Area of residence^†^			
Local	226 (67.9)	279 (84.3)	565 (85.9)
Not Local	107 (32.1)	52 (15.7)	93 (14.1)

Registered area of residence			
Urban	214 (64.3)	202 (61.0)	354 (53.8)
Rural	114 (34.2)	125 (37.8)	239 (36.3)
Pasturelands	5 (1.5)	4 (1.2)	65 (9.9)

Disease/health problem			
Cardiovascular and blood	87 (26.1)	81 (24.5)	166 (25.2)
Musculoskeletal and injuries	74 (22.2)	81 (24.5)	173 (26.3)
Genitourinary system and gestation	55 (16.5)	67 (20.2)	115 (17.5)
Respiratory system	68 (20.4)	46 (13.9)	86 (13.1)
Other	33 (9.9)	15 (4.5)	28 (4.3)
Digestive system	3 (0.9)	17 (5.1)	24 (3.6)
Eyes, ears, and skin	4 (1.2)	3 (0.9)	29 (4.4)
Metabolism, nervous system, and mental health problems	4 (1.2)	16 (4.8)	11 (1.7)
Cancer	3 (0.9)	4 (1.2)	20 (3.0)
Newborn and congenital anomaly	0 (0.0)	1 (0.3)	4 (0.6)
Infectious diseases	2 (0.6)	0 (0.0)	2 (0.3)

Symptoms			
Fever / Cough	77 (23.2)	62 (18.7)	80 (12.2)
Pain	124 (37.2)	110 (33.1)	268 (40.7)
Palpitations	28 (8.4)	23 (6.9)	53 (8.1)
Others	84 (25.2)	118 (35.7)	220 (33.4)
None	20 (6.0)	18 (5.4)	37 (5.6)

Repeat symptom			
Yes	165 (49.5)	177 (53.5)	292 (44.4)
No	168 (50.5)	154 (46.5)	366 (55.6)

Duration of symptoms			
≤3 days	105 (31.5)	120 (36.3)	222 (33.7)
4-30 days	133 (39.9)	120 (36.3)	252 (38.3)
>30 days	95 (28.6)	91 (27.4)	184 (28.0)

Reason for choosing hospital			
Distance	75 (22.5)	80 (24.2)	233 (35.4)
Finance	16 (4.8)	22 (6.6)	38 (5.8)
Quality	210 (63.1)	172 (52.0)	309 (46.9)
Attitude/Trust/Others	32 (9.6)	57 (17.2)	78 (11.9)

Patient status			
New patient	224 (67.3)	178 (53.8)	444 (67.5)
Established patient	109 (32.7)	153 (46.2)	214 (32.5)

^a†^Relative to the hospital where the patient sought treatment.

^b^WM: Western medicine; TCM: Traditional Chinese medicine; TMM: Traditional Mongolian medicine. UEBMIS: Urban Employees Basic Medical Insurance System; URBMIS: Urban Residence Basic Medical Insurance System; NRCMIS: New Rural Cooperative Medical Insurance System; SD: standard deviation.

**Table 4:** Final linear regression models presenting factors associated with expectation, perception, and gap (N=1,322).

Factor	Expectation			Perception			Gap		
	Adj. coeff. (95% CI)	P value (t-test)	P value (F-test)	Adj. coeff. (95% CI)	P value (t-test)	P value (F-test)	Adj. coeff. (95% CI)	P value (t-test)	P value (F-test)

Type of medicine: ref.=TMM			< 0.001			< 0.001			< 0.001
WM	-0.16 (-0.28,-0.05)	0.006		-0.66 (-0.84,-0.47)	< 0.001		-0.50 (-0.67,-0.32)	< 0.001	
TCM	-0.28 (-0.38,-0.18)	< 0.001		-0.05 (-0.22,0.11)	0.517		0.21 (0.05,0.36)	0.009	
Level of hospital: ref.=Provincial			< 0.001			< 0.001			< 0.001
City	-0.12 (-0.23,-0.02)	0.023		0.23 (0.06,0.40)	0.009		0.31 (0.15,0.47)	< 0.001	
County	-0.53 (-0.66,-0.41)	< 0.001		-0.12 (-0.32,0.08)	0.231		0.35 (0.17,0.52)	< 0.001	

^a^TMM: Traditional Mongolian medicine. TCM: Traditional Chinese medicine. WM: Western medicine.

^b^Note: The expectation model was adjusted for marital status, occupation, insurance status, monthly family income, duration of symptoms, and patient status.

The perception model was adjusted for nationality, marital status, occupation, insurance status, monthly family income, residential status, type of disease, duration of symptoms, and reason for choosing the hospital.

The gap model was adjusted for nationality, education level, monthly family income, current residence, duration of symptoms, reason for choosing the hospital, and patient status.

## Data Availability

The data used to support the findings of the system review in this study were provided by Health Information Reporting System (HIRS) of Inner Mongolia Autonomous Region under license, and so cannot be made freely available. Access to these data will be considered by the author upon request, with permission of Health and Family Planning Commission of the Inner Mongolia. The data used to support the findings of the survey in this study were supported by the Key Research Base of Humanities and Social Science of Inner Mongolia Autonomous Region, and so cannot be made freely available. Access to these data will be considered by the author upon request, with permission of Health Management School of Inner Mongolia Medical University.

## Additional Points

*Limitations of This Study*. The study was based on a small sample of hospitals of Inner Mongolia. Domination of females in our sample indicated a higher proportion of them using health services but may also imply an underrepresentation of males in the sample.

## Supplementary Materials

Supplementary 1 Supplementary Appendix  1: key informants interview guideline (english version).

Supplementary 2 Supplementary Appendix  2: patient survey questionnaire (english version).
