# Supplementary material for: Traditional Mongolian, Traditional Chinese, and Western Medicine Hospitals: System Review and Patient Survey on Expectations and Perceptions of Quality of Healthcare in Inner Mongolia, China
Source: Evid Based Complement Alternat Med. 2018 Jul 19;2018:2698461. doi: 10.1155/2018/2698461 (PMC6077555; doi:10.1155/2018/2698461)
Supplement: Supplementary 1 — Supplementary Appendix 1: key informants interview guideline (english version). [file 2698461.f1.docx]

Appendix 1:

Key Informants Interview Guideline (English Version)

Guideline 1: For Provincial Health Department

Interviewee: Department of TMM, Provincial health administrative department

Interviewee’s basic information:

Name: ________ Position: __________ Years of this position: _______

Theme

1 How about the history of TMM development in Inner Mongolia. Please give us a short introduction?

2 What is the train of thought of ​​the TMM development? Is there any develop plan for TMM? If yes, please give us some details.

3 Please introduce the basic situation of TMM this year. (Key emphasis in work, achievements, experiences, problems)

4 How about the policies about TMM development? Is there any representative policy? (System construction, Administrative mechanism, hospitals, human resources, qualifications, project, appropriate technology, industrial development)

5 How about the health service which providing by TMM? How many TMM hospitals are there in Inner Mongolia? How about the level of these hospitals?

6 What is the development situation of TMM industry?

7 How about the TMM management system? What are the functions of each department respectively?

8 How about the financial support of TMM development in Inner Mongolia? Is there any special funding from higher levels of government? Is there any special funding from this level of government?

9 How about the basic situation of health human resources for TMM?

10 Please introduce the linkage with international of medical service organization and system.

11 Is there any new method or conduction which can encourage the development of TMM? Please introduce.

12 In your opinion, what are the main problems facing by TMM development in Inner Mongolia? Do you have any recommendation?

Guideline 2: For Municipal Health Department

Interviewee：Department of TMM，Municipal health administrative department

Interviewee’s basic information:

Name: ________ Position: __________ Years of this position: _______

Theme

1 How about the history of TMM development in this city. Please give us a short introduction?

2 What is the train of thought of ​​the TMM development? Is there any develop plan for TMM in our city? If yes, please give us some details.

3 Please introduce the basic situation of TMM in this city this year. (Key emphasis in work, achievements, experiences, problems)

4 How about the policies about TMM development? Is there any representative policy? (System construction, Administrative mechanism, hospitals, human resources, qualifications, project, appropriate technology, industrial development)

5 How about the health service which providing by TMM? How many TMM hospitals are there in Inner Mongolia? How about the level of these hospitals?

6 What is the development situation of TMM industry?

7 How about the TMM management system? What are the functions of each department respectively?

8 How about the financial support of TMM development in Inner Mongolia? Is there any special funding from higher levels of government? Is there any special funding from this level of government?

9 How about the basic situation of health human resources for TMM?

10 Please introduce the linkage with international of medical service organization and system.

11 Is there any new method or conduction which can encourage the development of TMM? Please introduce.

12 In your opinion, what are the main problems facing by TMM development in Inner Mongolia? Do you have any recommendation?

Guideline 3: For Provincial Medical Educational Department

Interviewee：Department of TMM Education，Provincial Medical Educational Department

Interviewee’s basic information:

Name: ________ Position: __________ Years of this position: _______

Theme

1 How about the history of TMM educational system in our province. Please give us a short introduction?

2 What is the train of thought of ​​the development of TMM educational system? Is there any develop plan for TMM educational system in our province? If yes, please give us some details.

3 What the main contents of education of TMM? (Education with record of formal schooling, [Continuation education](http://dict.cn/continuation%20education), Teaching by master) How about related policies?

4 Please introduce the basic situation of TMM education in our province this year. (Number of medical school, Number of college major, Enrollment per year, Graduation per year, Employment of the students)

5 How about the situation of TMM training?

6 How about the financial support of TMM educational development in Inner Mongolia? Is there any special funding from higher levels of government? Is there any special funding from this level of government?

7 How about the basic situation of human resources for TMM education? What is the practice in the introduction of educational talent?

8 Is there any new method or conduction which can encourage the development of TMM education? Please give some introduction.

9 Please introduce the linkage with international of medical service organization and TMM schools or universities. What kind of cooperation?

10 In your opinion, what are the main problems facing by TMM educational development in Inner Mongolia? Do you have any recommendation?

11 In your opinion, what are the main problems facing by TMM development in Inner Mongolia? Do you have any recommendation?

Guideline 4: For Municipal Medical Educational Department

Interviewee：Department of TMM Education，Municipal Medical Educational Department

Interviewee’s basic information:

Name: ________ Position: __________ Years of this position: _______

Theme

1 What is the train of thought of ​​the development of TMM educational system? Is there any develop plan for TMM educational system in your city? If yes, please give us some details.

2 What the main contents of education of TMM? (Education with record of formal schooling, [Continuation education](http://dict.cn/continuation%20education), Teaching by master) How about related policies?

3 Please introduce the basic situation of TMM education in our province this year. (Number of medical school, Number of college major, Enrollment per year, Graduation per year, Employment of the students)

4 How about the situation of TMM training?

5 How about the financial support of TMM educational development in Inner Mongolia? Is there any special funding from higher levels of government? Is there any special funding from this level of government?

6 How about the basic situation of human resources for TMM education? What is the practice in the introduction of educational talent?

7 Is there any new method or conduction which can encourage the development of TMM education? Please give some introduction.

8 Please introduce the linkage with international of medical service organization and TMM schools or universities. What kind of cooperation?

9 In your opinion, what are the main problems facing by TMM educational development in Inner Mongolia? Do you have any recommendation?

10 In your opinion, what are the main problems facing by TMM development in Inner Mongolia? Do you have any recommendation?

Guideline 5: For Medical School or University

Interviewee：Dean of TMM Medical School or university

Interviewee’s basic information:

Name: ________ Position: __________ Years of this position: _______

Theme

1 What is the train of thought of ​​the development of TMM educational in your school or university? Is there any develop plan for TMM education? If yes, please give us some details.

2 What the main majors in your school? (Educational objective, [Courses](http://dict.cn/continuation%20education), Specialized development, Subject development)

3 What is the situation about the student of TMM in your school? (Enrollment、Major, Courses, Graduation, Employment status)

4 What are the main contents including in the TMM subjects development? What are the policies?

5 How about the situation of TMM educational development?

6 How about the development of TMM educational personnel?

7 Please introduce the linkage with international hospitals or medical schools or universities. What kind of cooperation?

8 In your opinion, what are the main problems facing by TMM educational development in Inner Mongolia? Do you have any recommendation?

9 In your opinion, what are the main problems facing by TMM development in Inner Mongolia? Do you have any recommendation?

Guideline 6: For Teachers in Medical School or University

Interviewee：Teachers in medical school or university

Interviewee’s basic information:

Name: ________ Position: __________ Years of this position: _______

Theme

1 What the main majors in your school? (Educational objective, [Courses](http://dict.cn/continuation%20education), Specialized development, Subject development)

2 What is the situation about the student of TMM in your school? (Enrollment、Major, Courses, Graduation, Employment status)

3 What is the situation about the text book about TMM?

4 What are the main procedures in the TMM teaching management?

5 How about the performances of TMM students? What is their feedback for the courses?

6 In your opinion, what are the main problems facing by TMM educational development? Do you have any recommendation?

7 In your opinion, what are the main problems facing by TMM development in Inner Mongolia? Do you have any recommendation?

Guideline 7: For Dean of TMM hospitals

Interviewee：Dean of TMM hospitals

Interviewee’s basic information:

Name: ________ Position: __________ Years of this position: _______

Theme

1 Is there any develop plan for TMM? If yes, please give us some details.

2 How about the policy focuses on the TMM hospitals (finance, admittance, service price, management, intendance )? Is there any special policy for TMM hospitals?

3 How about health service in this hospital? What the outpatient rate of the hospitals? How about the surgery?

4 If you just have some department providing the TMM health service, please give some introduction about the department. (Number of department, health service they providing)

5 Is there any special policy for these department or personnel in your hospital? (finance, management, service standard, human resources)

6 How many TMM human workers, including doctors, nurses and other technologist, in your hospital? How about their educational level and distribution in this hospitals?

7 How many training are there in your hospitals in the last year for TMM? (Plan, times, duration, contents, evaluation)

8 How about the situation of research on TMM in hospitals? (Projects, papers, books, patent treatment or medicine)

9 Do you have any linkage or cooperation with international TMM hospitals or organization in other provinces? If yes, what are the main contents of cooperation?

10 In your opinion, what are the main problems of the development of your hospitals? How to solve these problems? What kinds of support do you need?

11 In your opinion, what are the main problems facing by TMM development in Inner Mongolia? Do you have any recommendation?

Guideline 8: For Doctor of TMM hospitals

Interviewee：Doctors of TMM hospitals

Interviewee’s basic information:

Name: ________ Position: __________ Years of this position: _______

Theme

1 How did you become to be a TMM doctor? You learn TMM in university or family heritage?

2 Are you local people, or come from other place? How did you come to work in this hospital?

3 Are you satisfied with your work? (Income and benefit, workload, work circumstance, house, opportunity to grow and develop) Why?

4 How do you think about your work pressure? Is it high or low? Is your work time long or short? How about your relationship with patient?

5 How do you think about the patient's recognition of TMM doctor and treatment? In your opinion, why patients seek medical treatment of TMM?

6 Have you ever participant in any training in the last year? If yes, please give us some details. (Date, place, duration, contents, form, evaluation)

7 Have you got the certificate from government? What is your position? How do you think about your career prospect?8 Have you ever think about changing your position or hospitals before? If yes, why do you get that idea? What’s your plan about your career development in the future?

9 Do you know the policies about the TMM? Are these policies related to you? How do you think about these policies?

10 How about the performance appraisal system in hospitals? How do you feel about the policy?

11 Does your hospital have any special policy to encourage the development of TMM? If yes, how about the effect?

12 In your opinion, what are the main problems of the development of your hospitals? How to solve these problems? What kinds of support do you need?

13 In your opinion, what are the main problems facing by TMM development in Inner Mongolia? Do you have any recommendation?
