# Supplementary material for: Traditional Mongolian, Traditional Chinese, and Western Medicine Hospitals: System Review and Patient Survey on Expectations and Perceptions of Quality of Healthcare in Inner Mongolia, China
Source: Evid Based Complement Alternat Med. 2018 Jul 19;2018:2698461. doi: 10.1155/2018/2698461 (PMC6077555; doi:10.1155/2018/2698461)
Supplement: Supplementary 2 — Supplementary Appendix 2: patient survey questionnaire (english version). [file 2698461.f2.docx]

Appendix 2:

Patient Survey Questionnaire (English version)

ID：□□□□□□□□

**Characteristic of Hospital**

Name of the hospital:_____________________________ hid[ ][ ]

Inner Mongolia, China

**Characteristic of Clinic:**

Name of the clinic:_____________________________ cid[ ][ ]

**Interviewer (Signature) :** interview [ ][ ]

Date of interview: _________________

**Supervisor (Signature)：**

Date of check: _________________

| **Part I. Demographic Characteristic of Outpatient** | | |
| --- | --- | --- |
| **Variable** | **Specification** | **Data** |
| 1.1. Gender | [1] Male [2] Female | psex [ ] |
| 1.2. Date of birth | What year were you born? | pdobd [ ][ ]  pdobm [ ][ ]  pdob**y** [ ][ ][ ][ ] |
| 1.3 Nationality | ________ | pnat [ ] |
| 1.4.Respondent’s completed education level | [1] No formal schooling  [2] Primary school  [3] Middle school  [4] High school  [5] Diploma  [6] Graduate / Postgraduate | pedu [ ] |
| 1.5.Respondent’s marital status | [1] Single  [2] Married/ De Facto marriage  [3] Separated [4] Divorced  [5] Widowed  [6] Other ____________ | pmarr [ ] |
| 1.6. Respondent’s main occupation (By time) | [1] Production staff  [2] Transport staff  [3] Staff or servicer in company  [4] Staff in officer  [5] Technician  [6] Teacher  [7] President of government or institution  [8] Student  [9] Soldiery  [10] No occupation  [11] Others (Please specify) ____________  [12] Retired | pocc [ ][ ] |
| 1.7 Respondent’s Health insurance | [1] FMS: Free Medical Service  [2]UEBMI: Urban employee basic medical insurance  [3] URBMI: Urban residents basic medical insurance  [4] NRCMS : New Rural Co-operative Medical System  [5] CHI: Commercial medical insurance  [6] Others | pins[ ] |
| 1.8. Respondent’s Monthly family income(RMB) | [1] No income  [2] lower than 2000  [3] 2000-4000  [4] 4001-6000  [5] 6001-8000  [6] more than 8001 | pinc [ ] |
| 1.9. Respondent’s currently residence | [1] Local place  [2] Not local place | pliv [ ] |
| 1.10. Respondent’s [registered residence](http://dict.cn/registered%20residence) | [1] Urban area  [2] Rural area  [3] Pasturing area | preg[ ] |

| **Part II. Patients’ information about disease** | | |
| --- | --- | --- |
| **Variable** | **Specification** | **Data** |
| 2.1 What are your symptoms? | [1]Fever  [2] Pain  [3] Diarrhea  [4] Cough  [5] Palpitations  [6]Others  [7]None | psym [ ] |
| 2.2 Have you ever have the same symptoms before? | [1]Yes  [2] No | psym.b [ ] |
| 2.3 How many days have the symptom last? | ________ days | pdur [ ] |
| 2.4 Why do you choose this hospital? | [1] Convenience  [2] fair price  [3] High technical level  [4] Good facilitate  [5] Rich Drug  [6] Good Attitude  [7] Reimbursement fixed-point units  [8] Acquaintances  [9] Have trusted doctors  [10]Others | prea[ ] |
| 2.5 How long have you wait for seeing the doctor? | ________ hours | pwait [ ] |
| 2.6 What’s your diagnosis from doctor? (Please show us the diagnosis results) | ________ | pdiag [ ] |
| 2.7 Code of disease. | ________ | pcode [ ] |
| 2.8 Have you got any physical examination? Please write down. | ________ | pexam [ ] |
| 2.9 Have you got any treatment? | [1]Oral drugs  [2]Intramuscular injection  [3] Transfusion therapy  [4] Physical therapy  [5]Others (Please specify) ________ | ptreat [ ] |
| 2.10 How about the therapeutic outcome? | [1]Recovery  [2]Remission  [3] Has not yet started treatment  [4] Worsen  [5]Others (Please specify) ________ | pout [ ] |
| 2.11 Is this the first time you seek for your disease, or subsequent visit? | [1]First time (Turn to Question 2.14)  [2] Subsequent visit | pfir [ ] |
| 2.12 If this is your subsequent visit for this disease, why? | [1]Regular subsequent visit  [2] Doctors’ request  [3] Recrudescence  [4] Remaining problem  [5]Others (Please specify) ________ | psub [ ] |
| 2.13 How many days from your last visit to today’s subsequent visit? | ________ days | pday [ ] |
| 2.14 How much have you pay for this treatment? (RMB) | ________ Yuan | pcost [ ] |
| 2.15 Do these expenses can be reimbursed? | [1]Yes  [2] No (Turn to Question 2.14) | preim [ ] |
| 2.16 If expenses can be reimbursed, how much? (RMB) | ________ Yuan | preim.cost [ ] |
| 2.17 If expenses can be reimbursed, how? | [1] Abate directly  [2] Pay by self then reimburse in the hospital  [3] Pay by self then reimburse in fixed department | preim.how [ ] |
| 2.18 How much have you pay for other expenses? (Travelling, lodgment, purveyance ) (RMB) | ________ Yuan | pothexp [ ] |

Part III.

Directions

1 This part of questions is to investigate your expectation level of health service quality, not the quality of service you perceived actually in this hospital. If you have relatively high expectation, please select a higher score. If you don’t think the item is important, please choose a lower score.

2 Select the scores for each question on the base of your own judgment. The score will be designed from 1-9, which representing that "very low" (Score 1) to "very high" (Score 9), low (Score 3), general (Score 5), high (Score 7). Score 2,4,6,8 means the level between other two neighbors. Please select one answer for each question by circling on the corresponding value.

3 Please do not discuss with others.

| **Part III. Expectation of patients on health care** | | |
| --- | --- | --- |
| **Variables** | **Expectation of the service quality**  **Very low [1] [9] Very high** | |
| 3.1 Comfortable and convenient facilities | [ 1 ][ 2 ][ 3 ][ 4 ][ 5 ] [ 6 ][ 7 ][ 8 ][ 9 ] | e1[ ] |
| 3.2 Doctors and staff are always neat. | [ 1 ][ 2 ][ 3 ][ 4 ][ 5 ] [ 6 ][ 7 ][ 8 ][ 9 ] | e2[ ] |
| 3.3 Clean, comfortable  environment. | [ 1 ][ 2 ][ 3 ][ 4 ][ 5 ] [ 6 ][ 7 ][ 8 ][ 9 ] | e3[ ] |
| 3.4 Rational distribution of the service windows | [ 1 ][ 2 ][ 3 ][ 4 ][ 5 ] [ 6 ][ 7 ][ 8 ][ 9 ] | e4[ ] |
| 3.5 Meals are tasty and adapted to patients’ nutritious needs | [ 1 ][ 2 ][ 3 ][ 4 ][ 5 ] [ 6 ][ 7 ][ 8 ][ 9 ] | e5[ ] |
| 3.6 Patients feel secure in receiving medical care | [ 1 ][ 2 ][ 3 ][ 4 ][ 5 ] [ 6 ][ 7 ][ 8 ][ 9 ] | e6[ ] |
| 3.7 Doctors have a wide spectrum of knowledge and are competent | [ 1 ][ 2 ][ 3 ][ 4 ][ 5 ] [ 6 ][ 7 ][ 8 ][ 9 ] | e7[ ] |
| 3.8 Prompt service is provided to patients | [ 1 ][ 2 ][ 3 ][ 4 ][ 5 ] [ 6 ][ 7 ][ 8 ][ 9 ] | e8[ ] |
| 3.9 Patients should be treated with dignity and respect | [ 1 ][ 2 ][ 3 ][ 4 ][ 5 ] [ 6 ][ 7 ][ 8 ][ 9 ] | e9[ ] |
| 3.10 Doctors/staff listen to patients and keep them informed | [ 1 ][ 2 ][ 3 ][ 4 ][ 5 ] [ 6 ][ 7 ][ 8 ][ 9 ] | e10[ ] |
| 3.11 Doctors/staff should have morality in the work | [ 1 ][ 2 ][ 3 ][ 4 ][ 5 ] [ 6 ][ 7 ][ 8 ][ 9 ] | e11[ ] |
| 3.12 Doctors/staff should explain thoroughly medical conditions to patients | [ 1 ][ 2 ][ 3 ][ 4 ][ 5 ] [ 6 ][ 7 ][ 8 ][ 9 ] | e12[ ] |
| 3.13 Special treatment should get informed consents | [ 1 ][ 2 ][ 3 ][ 4 ][ 5 ] [ 6 ][ 7 ][ 8 ][ 9 ] | e13[ ] |
| 3.14 Details of charges should show clearly | [ 1 ][ 2 ][ 3 ][ 4 ][ 5 ] [ 6 ][ 7 ][ 8 ][ 9 ] | e14[ ] |
| 3.15 Doctors/staff should always willing to explain for patients about disease | [ 1 ][ 2 ][ 3 ][ 4 ][ 5 ] [ 6 ][ 7 ][ 8 ][ 9 ] | e15[ ] |
| 3.16 Inpatients’ services are easily accessible | [ 1 ][ 2 ][ 3 ][ 4 ][ 5 ] [ 6 ][ 7 ][ 8 ][ 9 ] | e16[ ] |
| 3.17 Doctors/staff are always willing to help patients | [ 1 ][ 2 ][ 3 ][ 4 ][ 5 ] [ 6 ][ 7 ][ 8 ][ 9 ] | e17[ ] |
| 3.18 Patients’ complaint are valued carefully | [ 1 ][ 2 ][ 3 ][ 4 ][ 5 ] [ 6 ][ 7 ][ 8 ][ 9 ] | e18[ ] |
| 3.19 Hospital services are easily accessible | [ 1 ][ 2 ][ 3 ][ 4 ][ 5 ] [ 6 ][ 7 ][ 8 ][ 9 ] | e19[ ] |
| 3.20 Patient privacy is protected | [ 1 ][ 2 ][ 3 ][ 4 ][ 5 ] [ 6 ][ 7 ][ 8 ][ 9 ] | e20[ ] |
| 3.21 Doctors/staff understand the specific needs of patients | [ 1 ][ 2 ][ 3 ][ 4 ][ 5 ] [ 6 ][ 7 ][ 8 ][ 9 ] | e21[ ] |
| 3.22 Doctors/staff should have patient’s best interests at heart | [ 1 ][ 2 ][ 3 ][ 4 ][ 5 ] [ 6 ][ 7 ][ 8 ][ 9 ] | e22[ ] |
| 3.23 Doctors/staff should be chosen by patients | [ 1 ][ 2 ][ 3 ][ 4 ][ 5 ] [ 6 ][ 7 ][ 8 ][ 9 ] | e23[ ] |
| 3.24 Clear diagnostic results are easily accessible | [ 1 ][ 2 ][ 3 ][ 4 ][ 5 ] [ 6 ][ 7 ][ 8 ][ 9 ] | e24[ ] |
| 3.25 Satisfactory therapeutic effect are easily accessible | [ 1 ][ 2 ][ 3 ][ 4 ][ 5 ] [ 6 ][ 7 ][ 8 ][ 9 ] | e25[ ] |
| 3.26 Affordable charges for drugs are rendered | [ 1 ][ 2 ][ 3 ][ 4 ][ 5 ] [ 6 ][ 7 ][ 8 ][ 9 ] | e26[ ] |
| 3.27 Affordable charges for physical examinations are rendered | [ 1 ][ 2 ][ 3 ][ 4 ][ 5 ] [ 6 ][ 7 ][ 8 ][ 9 ] | e27[ ] |
| 3.28 Affordable charges for foods and lodgment are rendered | [ 1 ][ 2 ][ 3 ][ 4 ][ 5 ] [ 6 ][ 7 ][ 8 ][ 9 ] | e28[ ] |

3.29 Your totally expectation on the health service quality of this hospitals is (please circle on the corresponding value)


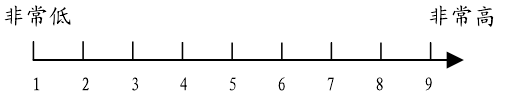


**Very low [1] Very high [9]**

3.30 In addition to the above aspects, what do you think we can use to evaluate the quality of health services:

Part IV.

Directions

1 This part of questions is to investigate your perception level of health service quality. Please show us your real feelings and real opinion to us. All your information will be keep in confidential.

2 Select the scores for each question on the base of your own judgment. The score will be designed from 1-9, which representing that "very low" (Score 1) to "very high" (Score 9), low (Score 3), general (Score 5), high (Score 7). Score 2,4,6,8 means the level between other two neighbors. Please select one answer for each question by circling on the corresponding value.

3 Please do not discuss with others.

| **Part IV. Perception of patients on health care** | | |
| --- | --- | --- |
| **Variables** | **Perception of the service quality**  **Very low [1] [9] Very high** | |
| 4.1 Comfortable and convenient facilities | [ 1 ][ 2 ][ 3 ][ 4 ][ 5 ] [ 6 ][ 7 ][ 8 ][ 9 ] | p1[ ] |
| 4.2 Doctors and staff are always neat. | [ 1 ][ 2 ][ 3 ][ 4 ][ 5 ] [ 6 ][ 7 ][ 8 ][ 9 ] | p2[ ] |
| 4.3 Clean, comfortable environment. | [ 1 ][ 2 ][ 3 ][ 4 ][ 5 ] [ 6 ][ 7 ][ 8 ][ 9 ] | p3[ ] |
| 4.4 Rational distribution of the service windows | [ 1 ][ 2 ][ 3 ][ 4 ][ 5 ] [ 6 ][ 7 ][ 8 ][ 9 ] | p4[ ] |
| 4.5 Meals are tasty and adapted to patients’ nutritious needs | [ 1 ][ 2 ][ 3 ][ 4 ][ 5 ] [ 6 ][ 7 ][ 8 ][ 9 ] | p5[ ] |
| 4.6 Patients feel secure in receiving medical care | [ 1 ][ 2 ][ 3 ][ 4 ][ 5 ] [ 6 ][ 7 ][ 8 ][ 9 ] | p6[ ] |
| 4.7 Doctors have a wide spectrum of knowledge and are competent | [ 1 ][ 2 ][ 3 ][ 4 ][ 5 ] [ 6 ][ 7 ][ 8 ][ 9 ] | p7[ ] |
| 4.8 Prompt service is provided to patients | [ 1 ][ 2 ][ 3 ][ 4 ][ 5 ] [ 6 ][ 7 ][ 8 ][ 9 ] | p8[ ] |
| 4.9 Patients should be treated with dignity and respect | [ 1 ][ 2 ][ 3 ][ 4 ][ 5 ] [ 6 ][ 7 ][ 8 ][ 9 ] | p9[ ] |
| 4.10 Doctors/staff listen to patients and keep them informed | [ 1 ][ 2 ][ 3 ][ 4 ][ 5 ] [ 6 ][ 7 ][ 8 ][ 9 ] | p10[ ] |
| 4.11 Doctors/staff should have morality in the work | [ 1 ][ 2 ][ 3 ][ 4 ][ 5 ] [ 6 ][ 7 ][ 8 ][ 9 ] | p11[ ] |
| 4.12 Doctors/staff should explain thoroughly medical conditions to patients | [ 1 ][ 2 ][ 3 ][ 4 ][ 5 ] [ 6 ][ 7 ][ 8 ][ 9 ] | p12[ ] |
| 4.13 Special treatment should get informed consents | [ 1 ][ 2 ][ 3 ][ 4 ][ 5 ] [ 6 ][ 7 ][ 8 ][ 9 ] | p13[ ] |
| 4.14 Details of charges should show clearly | [ 1 ][ 2 ][ 3 ][ 4 ][ 5 ] [ 6 ][ 7 ][ 8 ][ 9 ] | p14[ ] |
| 4.15 Doctors/staff should always willing to explain for patients about disease | [ 1 ][ 2 ][ 3 ][ 4 ][ 5 ] [ 6 ][ 7 ][ 8 ][ 9 ] | p15[ ] |
| 4.16 Inpatients’ services are easily accessible | [ 1 ][ 2 ][ 3 ][ 4 ][ 5 ] [ 6 ][ 7 ][ 8 ][ 9 ] | p16[ ] |
| 4.17 Doctors/staff are always willing to help patients | [ 1 ][ 2 ][ 3 ][ 4 ][ 5 ] [ 6 ][ 7 ][ 8 ][ 9 ] | p17[ ] |
| 4.18 Patients’ complaint are valued carefully | [ 1 ][ 2 ][ 3 ][ 4 ][ 5 ] [ 6 ][ 7 ][ 8 ][ 9 ] | p18[ ] |
| 4.19 Hospital services are easily accessible | [ 1 ][ 2 ][ 3 ][ 4 ][ 5 ] [ 6 ][ 7 ][ 8 ][ 9 ] | p19[ ] |
| 4.20 Patient privacy is protected | [ 1 ][ 2 ][ 3 ][ 4 ][ 5 ] [ 6 ][ 7 ][ 8 ][ 9 ] | p20[ ] |
| 4.21 Doctors/staff understand the specific needs of patients | [ 1 ][ 2 ][ 3 ][ 4 ][ 5 ] [ 6 ][ 7 ][ 8 ][ 9 ] | p21[ ] |
| 4.22 Doctors/staff should have patient’s best interests at heart | [ 1 ][ 2 ][ 3 ][ 4 ][ 5 ] [ 6 ][ 7 ][ 8 ][ 9 ] | p22[ ] |
| 4.23 Doctors/staff should be chosen by patients | [ 1 ][ 2 ][ 3 ][ 4 ][ 5 ] [ 6 ][ 7 ][ 8 ][ 9 ] | p23[ ] |
| 4.24 Clear diagnostic results are easily accessible | [ 1 ][ 2 ][ 3 ][ 4 ][ 5 ] [ 6 ][ 7 ][ 8 ][ 9 ] | p24[ ] |
| 4.25 Satisfactory therapeutic effect are easily accessible | [ 1 ][ 2 ][ 3 ][ 4 ][ 5 ] [ 6 ][ 7 ][ 8 ][ 9 ] | p25[ ] |
| 4.26 Affordable charges for drugs are rendered | [ 1 ][ 2 ][ 3 ][ 4 ][ 5 ] [ 6 ][ 7 ][ 8 ][ 9 ] | p26[ ] |
| 4.27 Affordable charges for physical examinations are rendered | [ 1 ][ 2 ][ 3 ][ 4 ][ 5 ] [ 6 ][ 7 ][ 8 ][ 9 ] | p27[ ] |
| 4.28 Affordable charges for foods and lodgment are rendered | [ 1 ][ 2 ][ 3 ][ 4 ][ 5 ] [ 6 ][ 7 ][ 8 ][ 9 ] | p28[ ] |

4.29 Your totally perception on the health service quality of this hospitals is (please circle on the corresponding value)

**Very low [1] Very high [9]**


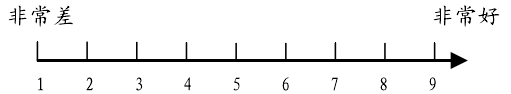


4.30 In addition to the above, is there any other aspects in your mind that the hospital give good service：

4.31 In addition to the above, is there any other aspects in your mind that the hospital should to improve：

4.32 In general, how do you satisfy with the health service this hospital provide to you？

[1] Strongly dissatisfied [2] Dissatisfied [3]General [4] satisfied [5]Strongly satisfied

4.33 If dissatisfied, have you ever complain to doctors?

[1]Yes [2] No (Turn to Question 4.35)

4.34 If you have ever complain to doctors, where did you complain

Please specify:

4.35 Will you come to this hospital to seek health service again?

[1]Yes [2] No

4.36 Will you introduce this hospital to other patient?

[1]Yes [2] No

*Thank you for your participation.*

*Finished time ____________________ (hr: min) finishh[ ] [ ] finishmm[ ] [ ]*
